# Bimanual training in stroke: How do coupling and symmetry-breaking matter?

**DOI:** 10.1186/1471-2377-11-11

**Published:** 2011-01-25

**Authors:** Rita Sleimen-Malkoun, Jean-Jacques Temprado, Laurent Thefenne, Eric Berton

**Affiliations:** 1 UMR 6233 Institut des Sciences du Mouvement E.J. Marey, CNRS & Université de la Méditerranée, 163 Avenue de Luminy, CP 910, 13288 Marseille, France; 2 HIA Laveran, Service de Réhabilitation Fonctionnelle, 13 Boulevard Laveran, 13013 Marseille, France

## Abstract

**Background:**

The dramatic consequences of stroke on patient autonomy in daily living activities urged the need for new reliable therapeutic strategies. Recently, bimanual training has emerged as a promising tool to improve the functional recovery of upper-limbs in stroke patients. However, who could benefit from bimanual therapy and how it could be used as a part of a more complete rehabilitation protocol remain largely unknown. A possible reason explaining this situation is that coupling and symmetry-breaking mechanisms, two fundamental principles governing bimanual behaviour, have been largely under-explored in both research and rehabilitation in stroke.

**Discussion:**

Bimanual coordination emerges as an active, task-specific assembling process where the limbs are constrained to act as a single unit by virtue of mutual coupling. Consequently, exploring, assessing, re-establishing and exploiting functional bimanual synergies following stroke, require moving beyond the classical characterization of performance of each limb in separate and isolated fashion, to study coupling signatures at both neural and behavioural levels. Grounded on the conceptual framework of the dynamic system approach to bimanual coordination, we debated on two main assumptions: 1) stroke-induced impairment of bimanual coordination might be anticipated/understood by comparing, in join protocols, changes in coupling strength and asymmetry of bimanual discrete movements observed in healthy people and those observed in stroke; 2) understanding/predicting behavioural manifestations of decrease in bimanual coupling strength and/or increase in interlimb asymmetry might constitute an operational prerequisite to adapt therapy and better target training at the specific needs of each patient. We believe that these statements draw new directions for experimental and clinical studies and contribute in promoting bimanual training as an efficient and adequate tool to facilitate the paretic upper-limb recovery and to restore spontaneous bimanual synergies.

**Summary:**

Since bimanual control deficits have scarcely been systematically investigated, the eventual benefits of bimanual coordination practice in stroke rehabilitation remains poorly understood. In the present paper we argued that a better understanding of coupling and symmetry-breaking mechanisms in both the undamaged and stroke-lesioned neuro-behavioral system should provide a better understanding of stroke-related alterations of bimanual synergies, and help clinicians to adapt therapy in order to maximize rehabilitation benefits.

## Background

Stroke is a major cause of functional disabilities and decrease in quality of life of people living in industrialized countries [1,2]. For instance, it is well established that mono-hemispheric cerebral vascular accident (CVA) results, among other diseases, in important chronic limitations of upper-limb use and manual dexterity [3,4] even after several months of rehabilitation [5,6]. In particular, it has been shown that CVA-induced spasticity [7,8] leads to significant slowing-down of unimanual movements, alteration of multijoint coordination [9], decrease in smoothness [10,11] and segmentation (multiple peak velocity) of reaching and grasping movements [12]. Moreover, diminished dexterity [13,14], decrease in muscular strength [15] and slowing [16] have also been observed in the (presumably intact) ipsi-lesional limb.

As most of our daily living activities require the use of both hands [17], disruptions of bimanual coordination induced by CVA lesions constitute an additional handicap for stroke patients. Thus, re-acquiring bimanual coordination is essential for making progress toward autonomy and should constitute a necessary objective for neuro-rehabilitation of upper-limb extremities [18]. However, in spite of well-identified neuro-physiological dependencies between limbs (e.g., [19,20]), for a long time, clinicians considered bimanual coordination as a simple addition of two uni-manual movements [21,22] and treated stroke-related alterations of bimanual synergies as the result of impaired capacities of the paretic limb. Thus, as noticed by McCombe Waller and Whitall [18], studies investigating CVA-induced impairment of bimanual coordination aimed primarily at analyzing movement kinematics and performance of the paretic limb (e.g., motor scores, dexterity index, qualitative functional tests) rather than between-limbs coordination.

Recently, bimanual movement training (BMT) has emerged as a complimentary tool in neuro-rehabilitation of the paretic upper-limb. BMT is a general term which includes multiple bilateral training techniques all of them requiring the simultaneous use of both upper-limbs in rehabilitation (see [23] for a review). Advanced justifications for BMT were grounded on the three main arguments: 1) the existence neurally-mediatted dependencies between limbs (see [19] for a review); 2) interhemispheric interactions along with the occurrence of bimanually triggered activation of similar neural distributed networks in both hemispheres [24-27]; and 3) evidence of training-related plasticity of the brain (e.g., [28-30]). The primary aim of BMT strategies has been to facilitate and enhance the recovery of the paretic limb. An additional argument was that bimanual training is closer than unimanual practice to every day tasks, which frequently requires coordinated use of both hands [18]. Accordingly, BMT is expected to maximize functional recovery through re-establishing both paretic limb control and bimanual control. However, in spite of the expected benefits, consistent findings regarding BMT's functional efficacy are still lacking and contradictory results have even been identified in the literature [18,23,26]. Thus, who could benefit from BMT and how it could be practically used in rehabilitation protocols remain largely unknown.

McCombe Waller and Whitall [18] suggested that more randomized controlled trials and neuro-physiological studies are still necessary to firmly confirm the efficacy of BMT for functional recovery of upper limb extremities in stroke patients. They hypothesized that inconsistent results observed in past studies on bimanual training could be attributed to the inadequate matching of protocols with baseline characteristics of the patients and the lack of functional assessment, which includes bimanual coordination tasks. In line with these statements, we suggest that the inadequate matching of BMT protocols with severity level of CVA lesions resulted mainly from: 1) the lack of evidence on how the mono-hemispheric lesion might perturb neurally-mediated dependencies between the limbs (which would alter inter-limb coupling strength, direction and signatures), 2) insufficient use of a variety of task constraints to account for patients' differences in neurally-mediated coupling between-limbs and symmetry breaking mechanisms. Indeed, quite similar bilateral tasks were used in all BMT protocols, independently of the specific characteristics of patients (see [23] for a review). Numerous studies carried out in the undamaged neuro-musculo-skeletal system (NMSS) have however demonstrated that bimanual synergies emerge by virtue of mutual coupling as an active, task-specific assembling process where the limbs are spontaneously or intentionally constrained to act as a single unit (e.g., [31,32]). Accordingly, exploring, assessing, re-acquiring and exploiting functional bimanual synergies following stroke require, as prerequisite, a deeper understanding of how the coalition of various constraints (i.e., neural, muscular, spatial, temporal, attentional,...) may facilitate/inhibit expressions of bimanual coupling at both neural and behavioural levels, as a function of the severity of CVA lesions. So, in order to adequately choose/adapt the bimanual rehabilitation protocol, one must take into account two fundamental variables: 1) the persistence (or not) of bimanual coupling following CVA, 2) the degree of the lesion-induced interlimb asymmetry. The degree of alteration of inter-limb coupling would determine whether the patient-specific BMT strategy should be directed toward mutual coupling rehabilitation (if altered) or whether the ipsi-lesional limb could be used as a "trainer" for the paretic one (via the persisting coupling). On the other hand, the degree of inter-limb asymmetry would determine how external constraints (task, environment) should be adapted in order to balance internal ones (lesion-induced), which will facilitate both the production of adaptive coordinated behaviour and the expression of the "positive" entrainment of the paretic limb by the "healthy" one.

In the present paper, we put the debate on the above rationale. Inspired by the conceptual framework of the dynamic system approach to bimanual coordination, we argue that neuro-behavioural manifestations of stroke-induced alterations of coupling and between-limb symmetry should be the cornerstone of both research and rehabilitation interventions. Accordingly, we draw two new directions for future experimental and clinical studies in the perspective of assessment and rehabilitation of upper-limbs in stroke patients: 1) behavioural consequences of CVA-induced impairment of bimanual coordination could be (at least in part) fruitfully explored by comparing, in join protocols, task-induced changes in coupling strength and asymmetry of bimanual discrete movements observed in both healthy people and stroke patients; 2) intervention protocols elaborated for restoring spontaneous bimanual coordination patterns that characterize the unimpaired NMSS (or aiming to exploit them) should be based on individual adaptation of task constraints in order to attenuate the stroke-related effects of decrease in bimanual coupling strength and/or increase in interlimb asymmetry.

## Discussion

### Coupling as a fundamental mechanism of bimanual coordination

In the undamaged NMSS, the motion of the limbs can be coordinated with seemingly unlimited temporal and spatial relationships, in either discrete or cyclical movements. Some of these patterns are spontaneously produced, while others require learning and intensive practice to be performed skilfully. For instance, in rhythmic movements, synchronized or alternated movements of limb components have been indentified as spontaneous coordination patterns [33-35]. In addition, it has been shown that spatial assimilation and synchronization tendencies between the two limbs can be intentionally overcome to perform bimanual coordination tasks with different (spatial and/or temporal) requirements [36-39]. Thus, both spontaneous and intentional/learned coordination patterns are the hallmark of the intact NMSS. Conversely, disruption of spontaneous coordination patterns of the bimanual repertoire can be considered as a signature of stroke-induced impairments in the NMSS.

During the last twenty years, theoretical foundations for a basic understanding of coordination principles and their neural basis have begun to emerge, thanks to the convergent contributions of nonlinear dynamic systems (e.g., [31,40]) and neural crosstalk (e.g., [30,41]) frameworks. In particular, research on interlimb coordination has shown that spontaneous coordination patterns emerge as the result of neurally-mediated cross-talks that occur at different levels of the central nervous system (CNS) facing internal (proper to the system) and external (environmental) constraints [31]. Mutual interactions between limb motions are captured by the abstract notion of coupling. Coupling may be thought as an informational flow substantiated through various substrates (neural, musculo-skeletal, spatial,... [42]), that links limb components. As the result of coupling, kinematic features of each hand motion are found in the trajectory of the other hand. For instance, when trying simultaneously to tap the head with one hand while rubbing the stomach with the other hand, each hand movement is reciprocally attracted to the other so that their trajectories become spatially and/or temporally coupled. Neural processes that mediate interlimb interactions can be accounted for by informational exchanges at the different levels of the CNS, resulting in neural crosstalk and finally, in kinematic coupling of limb trajectories [19,41,43,44]. For instance, as a result of neural crosstalk, activation of the muscles of one limb may affect muscle activity in the contralateral limb and then favour a specific and stable spatio-temporal relationship between limb motions [35,45]. In addition, coupling may be asymmetric, thereby leading to unequal influence of one limb to the other.

The most illustrative model system of this conceptual framework has been the study of phase transitions in bimanual, cyclical movement tasks [33-35]. In this situation, two spontaneous patterns were identified, corresponding to 0° and 180° of relative phase and then so-called "in-phase" and "anti-phase", respectively [35]. In the case of limb motion in the horizontal plane, the in-phase pattern involves symmetric motion of the hands in opposite directions, due to the simultaneous activation of homologous muscles, whereas the anti-phase pattern involves motion in the same direction, with simultaneous activity of antagonist muscles. The analysis of between limb coordination through the use of relative phase variability revealed different stability of the two patterns: The in-phase pattern proved to be more stable than the anti-phase pattern. Moreover, an unavoidable switch (transition) occurred from the latter to the former (i.e. a phase transition) when oscillation frequency increases beyond a given critical threshold. This behavioural picture of bimanual coordination has been called "spontaneous coordination dynamics" [31,33]. It depends essentially upon neural cooperation that expresses, at the behavioural level, the results of two competing forces within the CNS that would otherwise remain invisible. On the one hand, a tendency for limb components to adopt a specific, persisting behaviour (as a result of their intrinsic neuro-mechanical properties e.g. eigenfrequency), and on the other hand, a tendency for each limb component to be attracted toward the other (by virtue of coupling). Finally, when moving together in a bimanual task, limbs are constrained to operate as a single functional synergy, which is the observable outcome of a continuous struggle between maintenance and attraction within the NMSS.

One can easily figure out that if one or the two competing forces is/are modified as the result of internal (e.g., CVA lesion, mechanical factors), environmental (e.g., metronome) and/or task (e.g., movement frequency) constraints, both the bimanual repertoire and the transient capacity of assembling a task-specific relationship between limbs will be affected. Numerous works have confirmed this prediction in the undamaged neuro-behavioural system. For instance, it has been suggested that increase in movement frequency decreased coupling strength, giving rise to loss of stability of the actual pattern and, finally, to a switch toward a more attractive phase relation (e.g., [33]). Conversely, attentional focus has been shown to increase coupling strength, thereby stabilizing the actual pattern and delaying the occurrence of phase transition from anti-phase to in-phase [46,47]. At the individual component level, a change in natural movement frequency resulting from a modification of neuro-mechanical properties of one of the oscillating segment has been shown to create a difference between the two components, which offset the attraction toward perfect synchronization between them (e.g., [31,48]). To our knowledge, this conceptual framework has never been applied to stroke-related impairments of bimanual coordination (but see [49] for an introduction).

Coupling mechanisms and limitations of the CNS in mastering interlimb coordination were also revealed in discrete bimanual tasks (e.g., [39,50,51]). In this context, spontaneous tendencies to spatio-temporel synchronization of both limbs reflect what are the most easily potentiated pathways and neural cross-talk in the nervous system. For instance, when trying to draw a line and a circle simultaneously, one with each hand, amplitudes and directions tend to be similar so that the line is attracted toward the circle (it becomes elliptic) and, conversely, the circle is compressed and tend toward a line. This so-called spatial assimilation reflects, at a behavioural level, the effects of underlying neural coupling mechanisms. Similarly, drawing orthogonal lines in front of the body is more difficult than drawing parallels, thereby revealing underlying neural interference giving rise to directional coupling (see [30] for an overview).

For clinicians, a major implication of these findings is that the observed coordination patterns provide a window into underlying coupling mechanisms in the NMSS. Accordingly, stroke-induced alterations in bimanual coordination must be assessed through impairments of the relationship between limbs rather than through changes in the kinematics of individual limb components [18]. Evidence exists of CVA-induced impairment of bimanual control but knowledge about the alterations of neural coupling and how it can be influenced by task constraints in stoke patients remain limited.

### Limited knowledge is available about stroke-induced alterations of bimanual coupling

#### Cyclic bimanual movement tasks

Since Mudie & Matyas's seminal work [52,53], subsequent studies have explored inter-limb interactions in stroke patients and the effectiveness of bilateral arm training for rehabilitation of upper limb extremity (see [18,54] for reviews). It has been shown that stroke patients with severe lesions usually encounter great difficulties to perform cyclic movements even at a relatively low frequency [55]. Consequently, assessment of bimanual coordination dynamics is not practical with severely impaired patients. Studies carried out with mild impaired stroke patients showed that, as compared to healthy adults, patients were less stable and less accurate both in in-phase and in anti-phase coordination patterns (e.g. [56-58]). In continuous temporally symmetric movement Rice and Newell [58] showed that, in post-stroke individuals, the non-paretic limb was constrained to the slower paretic limb frequency and, consequently, was unable to achieve its unimanual natural frequency. Lewis and Byblow [56] examined interlimb temporal and spatial coordination in a continuous circle-drawing task in post-stroke hemiparetic individuals. Their results showed that the paretic limb influenced the behaviour of the non-paretic limb and no improvements in the paretic one were elicited with the continuous bimanual task. Ustinova *et al. *[59] investigated how the bilateral coordination pattern was regained after external perturbations of either the paretic and non-paretic limbs. Results showed that the relaxation time toward the initial anti-phase pattern was longer for stroke patients than for control participants (2 cycles versus 1 cycle, respectively). These findings suggested that coupling strength was altered, though weakly, in mildly impaired stroke patients.

#### Discrete bimanual movement tasks

Inconsistent results have been observed in the literature with respect to CVA effects on coupling in bimanual discrete coordination tasks. Some studies did not find kinematic signatures of coupling in either the paretic or non-paretic limb kinematics during bimanual movements (e.g., [60]). On the other hand, other experiments succeeded in demonstrating the persistence of symmetric coupling after CVA. In a group of moderately impaired patients, Harris-Love *et al. *[61] observed symmetric coupling interference on movement time (MT), peak velocity (PV), and peak acceleration (PA) of both arm trajectories, as well as on symmetry ratios for each variable (i.e. the value of the paretic arm divided by non-paretic one). Messier *et al. *[62] reported a similar pattern for elbow joint motion during simultaneous parallel bilateral movements. These results indicated the persistence of a symmetric coupling as usually observed in healthy participants. Finally, several studies showed an increase in asymmetric coupling after CVA. For instance, Dickstein *et al. *[63], investigating unilateral and bilateral arm movements resulting from elbow-joint mobilization, reported a prolonged movement time for the non-paretic limb during bilateral elbow flexion compared to the unilateral condition. Temporal asymmetrical coupling, in which the paretic limb slowed the non-paretic one, has also been reported in other studies [55,56,61,62,64,65]. On the other hand, Cunningham *et al. *[66] showed some facilitation of the paretic limb that is a smoother elbow extension velocity profile during bimanual movements.

Thus, though a number of studies confirmed that bimanual movements are disrupted in stroke patients, the above reviewed literature showed inconsistent results with respect to the stroke-induced alterations of bimanual coupling. A possible explanation is that in the different studies, bilateral movement tasks were proposed quite independently of the severity of CVA lesion and, consequently, might have sometimes hidden behavioural expression of interlimb coupling. This hypothesis is grounded on the theoretical premise that bimanual coordination in stroke results from the struggle between two competing forces: mutual attraction between limbs (depending on the strength of neural coupling) and symmetry-breaking (resulting from difference in neuro-mechanical properties of the limbs), which are both affected by CVA lesion. However, the balance of these two competing forces can also be modulated by task constraints.

### Bimanual coordination in stroke as an expression of the balance between coupling strength and symmetry-breaking between limbs

An attractive hypothesis concerning bimanual coordination stroke patients is that CVA lesion modifies both the coupling scheme within the neuro-behavioral system and the neuro-mechanical characteristics of individual limb components (i.e., the paretic and non paretic limbs). Evidence exists in the literature on the undamaged NMSS, that the balance between coupling strength and differences in neuro-mechanical limb properties determines interlimb (de)synchronization in discrete bimanual coordination tasks. The resulting symmetry-breaking refers to the offset of spatial and temporal synchronization of hand motions, A main source of symmetry-breaking in bimanual coordination tasks has been reported first by Kelso, Delcolle and Schöner ([67]; but see also [68]). It lies in intrinsic differences between limb properties, such as the difference in movement times or natural frequency of each limbs, and may lead to a variety of coordinative phenomena: From moderate shifts in relative timing between limbs, which perturbs the bimanual coordination and manifests either by a (more or less large) shift in between-limbs relationship or even by "relative coordination" in which limb movements are no longer (or only transiently) attracted to a stable coordination pattern.

Schöner [51] proposed a model to capture the synchronization/de-synchronization tendencies observed in discrete bimanual coordination in the undamaged neuro-behavioral system (see [49] for details). In this framework, relative timing between limbs expresses the underlying coordinative activity of the CNS. Accordingly, one predicts that depending on the magnitude of neuro-mechanical differences between limbs, bimanual coordination may (or not) be dominated by symmetry-breaking and coupling may (or not) be strong enough to lead to synchronization tendency. Thus, depending on the importance of decrease in coupling strength relative to the magnitude of difference between neuro-mechanical limb properties, patterns characterized by sequential initiation of discrete movements (i.e. initiation time, IT) and/or differences in movement time (MT) will be observed. This prediction accounted for experimental (and supposedly clinical) conditions in which the two limbs differed individually in their amplitude and/or movement duration [39,50,69,70]. An elegant illustration can be found in Kelso *et al.*'s study [39,50], where the authors manipulated task difficulty in unimanual and bimanual aiming tasks by varying both width and distance of the target. In unimanual conditions, as predicted by Fitts's law [71,72], MT increased with the index of difficulty (ID). In the bimanual conditions, task difficulty was manipulated either symmetrically (i.e. same ID for both hands) or asymmetrically (i.e. a different ID for each hand). Results showed that, when confronted to dissimilar movement task constraints, the fast hand slowed down to move synchronously with the slower hand, which was weakly affected by the faster one. These results suggest that there is a strong tendency for limbs to be synchronized even under conditions of disparate difficulty. However, Marteniuk *et al. *[69] and Corcos [70] also showed a tendency to a breakdown of synchronization when the intrinsic movement times (reflecting task demands) become too dissimilar, which means that coupling was not strong enough to maintain a synchronized bimanual synergy. Riek *et al. *[73] observed that increasing asymmetry between limbs also increased sequential initiation of movements: The hand performing the longer amplitude started before the hand performing the shorter amplitude but the two hands arrived on the target simultaneously.

This theoretical framework provides new insight to assess stroke-induced alterations of bimanual coordination and might help to define principles of therapeutic interventions dedicated to functional recovery of coordinated behaviour. These hypotheses rest however on the assumption that: 1) the NMSS becomes internally constrained in an asymmetrical manner by CVA lesions at both the level of coupling and individual components (e.g., spasticity), and that 2) a parallel can be drawn between symmetry breaking of bimanual coordination in healthy people and stroke-related alterations of coupling strength and limb properties. In the following section, we discuss how the study of the interplay between coupling and symmetry-breaking mechanisms could help researchers and clinicians to better understand bimanual coordination impairments in stroke patients and correspondingly better individually adapt their interventions in respect to the patient characteristics.

### Join protocols to study symmetry-breaking of bimanual coordination in the undamaged and in CVA-lesioned neuro-behavioral systems

Our main assumption is that, in a bilateral coordination task, shifts in relative timing between limbs express the results of counteracting effects of attraction and symmetry-breaking factors. These factors may originate in stroke-induced weakness of neural coupling between limbs and/or in difference in neuro-muscular stiffness between the paretic and non-paretic limbs (e.g., spasticity), respectively. Their effects on bimanual coordination can be experimentally investigated in both healthy and stroke patients. In this aim, a possible experimental strategy consists of the comparison of the observed behaviours of both populations in similar experimental paradigms and tasks conditions, though adapted to baseline characteristics of stroke patients. Kelso *et al.*'s experimental paradigm [39] might be a valuable a model system in this respect.

By using bimanual Fitts's task paradigm, temporal asymmetry between limbs can progressively scaled by changing either biomechanical (loading, muscular co-contraction) or tasks/informational constraints (e.g., Fitts's ID) applied to each limbs. Neural coupling can also be modulated by changing attentional focus directed to the task [47] or to a limb [74], or even by removing vision. According to loss of coupling and symmetry-breaking predictions, when increasing asymmetry between limbs, one should observe a progressive de-synchronization of IT and/or MT and finally, when temporal asymmetry between limbs goes beyond a critical threshold, an abrupt transition from synchronized movements to a desynchronized pattern. In the undamaged neuro-behavioral system, transitions should be preceded by an increase in variability of the difference in movement times between limbs. One also predict that, for a given magnitude of temporal asymmetry between limbs, if coupling strength is increased, one should observe: (a) a better synchronization for larger interlimb temporal asymmetries, and (b) a higher stability of synchronized patterns and less transition for higher magnitude of temporal asymmetry between limbs. These predictions are in large part deduced from disparate experimental facts (e.g., [39,50,69,73,75]), but to our knowledge they have never been tested systematically. In doing so, one could establish a reference frame for further analyzing bimanual asymmetries resulting from CVA lesions. Indeed, an important question is whether and in what conditions comparable behaviours will be observed in stroke patients and healthy people with respect to the effects of asymmetry between limbs, be it caused by mechanical, informational or attentional factors.

Following the same logic in stroke patients, bimanual asymmetry could be attenuated by manipulating movement time of the non-paretic arm, while keeping constant the difficulty of the task for the paretic arm. By doing so, one makes bilateral movements more symmetric, thereby increasing the effect of neural coupling and improving synchronization. Participants could also be instructed to focus their attention on the bimanual task or to the paretic limb to increase bimanual coupling and then to facilitate synchronization. Thus, one could determine whether (more or less severely impaired) stroke patients perform more synchronized bimanual patterns when asymmetry between limbs is attenuated by specific manipulations of constraints on either the non-paretic (e.g. progressive loading, manipulation of ID) or the paretic arm (focus of attention, reduction of spasticity). Moreover, it can be anticipated that transient loss of coordination between limbs, characterized by a complete independence of each limb at a given level of dissimilarity between limbs (i.e., relative coordination), could be more frequently observed in stroke patients. In this respect, the effects of reducing spasticity of the paretic limb on bimanual coordination will also be of particular interest to distinguish the respective effects of neuromuscular factors and neural coupling on the occurrence of relative coordination. For instance, a crucial question is whether entrainment and synchronization can be gained or whether de-synchronization can be delayed after reducing limb spasticity. Finally, one can hypothesize that, on the basis of this experimental paradigm, it could be possible to determine whether behavioural expressions of asymmetries and relative coordination caused by CVA lesions depend on the location in the dominant/non dominant hemisphere of the lesion, the age of the patient, the constraints of the task, etc.... To our knowledge, these predictions have never been empirically tested for discrete movement tasks in both healthy and stroke participants.

In addition to research studies, further application of the above "loss of coupling" and "symmetry-breaking" hypotheses to stroke rehabilitation could also be envisaged by introducing a patient-specific constraint-adapted BMT.

### Implications of coupling and symmetry-breaking for BMT in stroke

Following the same aforementioned reasoning, in order to facilitate the expression of neuro-behavioral coupling and make use of it in rehabilitation, it is necessary to adapt bimanual protocols to patient baseline characteristics. It can be done, in particular, by modulating task-constraints to be applied to non-paretic limb during bimanual rehabilitation. This recommendation is grounded on the above-mentioned coordination principles established in the undamaged NMSS. It could be however further explored in clinical studies on BMT.

As a preliminary consideration, it is necessary to clarify what the notion of coupling means in the context of bimanual rehabilitation. Commonly, since bilateral training is often used as a tool to help recover the paretic limb control, the bimanual coupling is considered as functional only when it permits to create a "positive" entrainment effect that is, when temporal and/or spatial features of the non-paretic arm trajectory interfere with the paretic arm trajectory. In other words, a main expectation in BMT is that the non-paretic limb would "entrain" the impaired one thereby improving its performance. As a result, in two-handed conditions, the paretic limb movement is expected to be faster, more accurate, and smoother then in one-handed ones. Not surprisingly, such prediction has rarely been verified in the experimental studies. From the theoretical point of view, coupling exists as long as there are spatial and temporal interferences between the limbs no matter the direction (i.e. from the paretic to the non-paretic and/or from the non-paretic to the paretic). Actually, even an asymmetric influence of the paretic limb toward the non-paretic indicates the persistence of bimanual coupling. Furthermore, results observed in both healthy (e.g. [70,75]) and stroke participants [55,56,61,62,64,65] rather suggest that a predominant asymmetric coupling effect, from the slower (i.e. paretic) to the faster (i.e. non paretic) limb, should be expected. Therapeutic interventions should then wisely choose the appropriate settings of bimanual training tasks in order to counterbalance the neuro-mechanical asymmetries between limbs thereby facilitate the expression of a more mutual inter-limb entrainment and to optimize the recovery of bimanual coordination by increasing coupling strength.

Presumably, both "abstract" training tasks (i.e. BATRAC^®^, [24]) and functional daily-living-inspired tasks could be successfully used in BMT protocols as long as the coupling and symmetry-breaking principles are correctly taken into account. The cornerstone for therapeutic interventions lies in the identification, appropriate setting and adequate manipulation of different external constraints (environmental and task-related). This could be made, for instance, by: (a) adapting mean movement time, (b) reducing effective asymmetry between limbs (e.g. by loading the arm, reducing spasticity,...), (c) balancing asymmetry through manipulating accuracy constraints, (d) changing attentional conditions either by instructing participants to allocate more attention to the paretic arm or by re-organizing the starting point and targets locations (see [73,76]). One can speculate that these modulations of external constraints will improve plastic changes at the different levels of the CNS. Therapists may expect to obtain both short-term and delayed effects on coordinated behaviour by conjugating: (a) scaling of control parameters of the coordination pattern (e.g. movement speed, physical support,...), (b) augmented behavioural information (e.g. instruction on movement goal, auditory or visual feedback, guiding metronome...) and (c) practicing single or multiple coordination task across multiple training sessions. Short-term effects would result from transient, intentional forcing of bimanual synergy to adapt to task constraints, while long-term effects would result from stabilized CNS adaptations (brain plasticity) incurred by repetitive practice in specific conditions.

To sum up, the important message for clinicians is that, for a given coordination pattern, inappropriate scaling of task constraints may preclude or, conversely, facilitate the production of an adaptive coordinated behaviour. In this respect, the attention of therapists should be drawn on several important points: (a) assessing the persistence of the basic repertoire of bimanual movements may be considered as a preliminary step indicating specific dysfunctions in the neuro-behavioral system; (b) gains in bimanual coordination do not automatically arise from progress of unimanual movements, instead, they must be trained as specific synergies, not as the sum of two single limbs; (c) restoring the default mode of coupling may be indicative of an ongoing re-learning process, which is of potential benefit for stroke patients; and (d) bilateral training is beneficial for the paretic limb only when bimanual coupling is unimpaired and inter-limb asymmetry is small (surmountable or partially compensated).

## Summary

Even after a long rehabilitation period, stroke patients continue to suffer from long-term impairments in their daily living activities. If properly conducted BMT could be an efficient approach to significantly reduce such handicap and accelerate the recovery of the upper-limbs function. In spite that bilateral therapy was classically used as an additional approach to restore the paretic limb control it should above all be looked at as predilection strategy to train bimanual behaviour. Since bimanual control deficits have scarcely been systematically investigated in the context of stroke, many uncertainties remain on the adequate prescription and the true value of BMT. Clearly, researchers and clinicians should pay a lot more interest to bimanual coordination assessment and rehabilitation. In the quest for evidence and guidelines concerning the appropriate use and settings of BMT protocols, "coupling" and "symmetry-breaking" are very promising concepts to guide researchers (randomized controlled research studies) and therapists (individualized rehabilitation protocols). New directions for research and intervention are put into debate in the present paper. However, only the results of future research and clinical studies will determine the evidence-based recommendation for therapy. Finally, one can argue that BMT might be a promising tool for neuro-rehabilitation, under the reserve that both researchers and clinicians develop specific approaches to assess, restore and exploit bimanual coupling and symmetry breaking phenomena.

## Competing interests

The authors declare that they have no competing interests.

## Authors' contributions

RSM elaborated the general structure of the manuscript and drafted it. JJT and LT critically revised the draft. EB supervises the research program. All authors have read and approved the final version of the manuscript.

## Authors' information

RSM: She is physiotherapists and PhD student in the Institute of Movement Science in Marseille. She has a long experience of classical upper-limb rehabilitation strategies. Her PhD research program aims at exploring CVA-induced alterations of coupling processes and neuro-mechanical properties of paretic and non-paretic limbs by comparing healthy people and stroke patients in both assessment and rehabilitation protocols.

JJT: He is full professor at the Institute of Movement Science at the University of the Mediterranean. He is a well-recognized specialist of the dynamical systems approach to bimanual coordination. During the last 10 years, he has published a number a paper exploring the role of the coalition of various constraints (muscular, directional, attentional,...) on bimanual coordination dynamics in the undamaged neuro-musuclo-skeletal system. In particular, he published reference papers on attention and bimanual coordination in the framework of the theory of dynamical systems (Monno *et al.*, 2000, Temprado *et al.*, 1999; for a review Monno *et al.*, 2002).

LT: He is medical doctor in physical medicine and rehabilitation. He is head of the department of functional rehabilitation in Laveran hospital. He supervises the rehabilitation protocol elaborated and tested by the authors in a currently on-going research program on bimanual rehabilitation, coupling and symmetry breaking.

EB: He is a specialist of movement biomechanics. He is the head director of the Institute of Movement Sciences where the research program is undertaken.

## Pre-publication history

The pre-publication history for this paper can be accessed here:

http://www.biomedcentral.com/1471-2377/11/11/prepub
